# *In Vivo* Quantification of Myocardial Infarction in Mice Using Micro-CT and a Novel Blood Pool Agent

**DOI:** 10.1155/2017/2617047

**Published:** 2017-10-16

**Authors:** Stefan Sawall, Danielle Franke, Anne Kirchherr, Jan Beckendorf, Jan Kuntz, Joscha Maier, Alexander Kraupner, Johannes Backs, Andreas Briel, Marc Kachelrieß

**Affiliations:** ^1^Medical Physics in Radiology, German Cancer Research Center (DKFZ), Im Neuenheimer Feld 280, 69120 Heidelberg, Germany; ^2^nanoPET Pharma GmbH, Luisencarrée, Robert-Koch-Platz 4, 10115 Berlin, Germany; ^3^Department of Molecular Cardiology and Epigenetics, University of Heidelberg, Heidelberg, Germany; ^4^German Centre for Cardiovascular Research (DZHK), Partner Site Heidelberg/Mannheim, Heidelberg, Germany

## Abstract

We herein developed a micro-CT method using the innovative contrast agent ExiTron™ MyoC 8000 to longitudinally monitor cardiac processes* in vivo *in small animals. Experiments were performed on healthy mice and mice with myocardial infarction inflicted by ligation of the left anterior descending artery. Time-dependent signal enhancement in different tissues of healthy mice was measured and various contrast agent doses were investigated so as to determine the minimum required dose for imaging of the myocardium. Due to its ability to be taken up by healthy myocardium but not by infarct tissue, ExiTron MyoC 8000 enables detection of myocardial infarction even at a very low dose. The signal enhancement in the myocardium of infarcted mice after contrast agent injection was exploited for quantification of infarct size. The values of infarct size obtained from the imaging method were compared with those obtained from histology and showed a significant correlation (*R*^2^ = 0.98). Thus, the developed micro-CT method allows for monitoring of a variety of processes such as cardiac remodeling in longitudinal studies.

## 1. Introduction

The introduction of micro-CT has brought about considerable improvements in anatomical imaging of small animals through the modality's high spatial resolution that enables identification of morphological changes in small structures. However, it is a fact that the acquisition time in typical micro-CT devices is significantly higher than those of clinical devices. Indeed, whereas a clinical scan may take even less than one second, a micro-CT scan requires around 30 min and, at times, may even take up to 1 h. Such long scan times necessitate the use of a contrast agent that is not rapidly cleared from the animal but, instead, persists in the blood for a prolonged period of time. Due to the rapid clearance of clinical CT contrast agents from the blood pool of small animals, clinical agents are not optimal for small animal imaging [1, 2]. In fact, when using clinical CT contrast agents in micro-CT studies, the data acquired is inconsistent and image reconstruction is only possible using sophisticated injection protocols [3]. To overcome the issue of rapid contrast agent clearance, micro-CT investigations are generally coupled with blood pool contrast agents, which persist in the blood for a prolonged period of time.

Currently, there are a handful of commercially available micro-CT blood pool agents, which find application in vessel imaging, liver and spleen imaging, and cardiac imaging [4–11]. In view of cardiac imaging, for example, in studies of cardiac infarction and remodeling, the prolonged blood circulation time of micro-CT blood pool agents renders assessment of morphological and functional cardiac parameters possible. Besides their long blood half-life, these agents preferably create a high contrast between the blood and the myocardium at a low injection volume. Due to the inherently low blood volume of small animals, low injection volumes are desired so as to minimize adverse haemodynamic effects. Furthermore, in cardiac micro-CT studies, the animal models, for example, model for myocardial infarction, tend to be susceptible to volume overload. Thus, for cardiac imaging, a blood pool agent with a high concentration of the signal-enhancing moiety is preferred, allowing for high contrast at low injection volume.

Owing to the high cardiac rate (about 600 beats/min) and rapid respiratory rate (about 300 breaths/min) of small animals, cardiac micro-CT requires the application not only of high-contrast blood pool agents but also of reconstruction methods that allow significant reduction of motion artifacts. While it is indisputable that cardiac parameters can be obtained using positron emission tomography (PET), single photon emission computed tomography (SPECT), or magnetic resonance imaging (MRI), micro-CT reconstruction methods provide images with superior spatial and temporal resolution [12–15].

In this study, we develop a micro-CT method using the innovative micro-CT blood pool agent ExiTron MyoC 8000, having a high iodine concentration (210 mg I/mL), to longitudinally monitor cardiac function in a mouse model of myocardial infarction. Through measurement of the infarct volumes observed on the micro-CT reconstructions and comparison with the volumes obtained from histopathology, we assess the feasibility of the method to noninvasively monitor cardiac processes and pathologies in small animals.

## 2. Materials and Methods

### 2.1. Animals

All animal experiments were approved by the local committee on animal welfare (G-34/11, G-6/13) and were in accordance with the guidelines issued by the Federation of European Laboratory Animal Science Associations (FELASA) [16]. The healthy animal group (*n* = 15) consisted of C57BL/6J mice having a mean body weight of 22 ± 3 g, originally purchased from Charles River (Sulzfeld, Germany) and bred in-house for at least 8 generations. The infarct animal group (*n* = 5) consisted of C57BL/6J mice inflicted by ligation of the left anterior descending artery (LAD), as previously described [17, 18]. Briefly, the surgical procedure entailed a thoracotomy between the 3rd and 4th ribs and partial removal of the pericardial sac. Following location of the LAD between the pulmonary artery and the left auricle, the LAD was ligated using an 8-0 Prolene suture (Ethicon, Norderstedt, Germany). After positioning of a chest tube between the 4th and 5th ribs, the thoracic cavity was closed in a layered manner. A postoperative period of 2 weeks was allowed before start of the imaging experiments.

### 2.2. Imaging

The innovative micro-CT contrast agent used in this study (ExiTron MyoC 8000, Viscover™, nanoPET Pharma GmbH, Berlin, Germany) is based on nanoparticles having a mean hydrodynamic diameter of 300 nm, prepared using an interfacial deposition method of a polymer followed by solvent displacement [19]. Compared to ExiTron nano 6000 and ExiTron nano 12000, which consist of solid nanoparticles of high alkaline-earth metal content, ExiTron MyoC 8000 is based on nanocapsules with a high iodine concentration (210 mg/mL). The white opaque dispersion is colloidally stable on the long term and biocompatible, with pH and osmolality values similar to those under normal physiological conditions.

To investigate contrast agent kinetics, healthy anaesthetized mice (*n* = 10) were first imaged prior to contrast agent injection. The mice were then injected with ExiTron MyoC 8000, at a dose of 1050 mg I/kg body weight (i.e., 125 *μ*L per 25 g mouse) followed by a saline flush (25 *μ*L). After injection of the contrast agent, micro-CT was performed on the animals repetitively over 250 min with an additional scan after 24 h using a prototype flat detector-based micro-CT system (Siemens Healthineers, Forchheim, Germany) [4]. The system provides a source-isocenter distance of 570 mm and an isocenter-detector distance of 360 mm, resulting in a spatial resolution in the center of rotation of 238 *μ*m, given a detector with a matrix size of 1024 × 192 pixels and a square pixel size of 388 *μ*m. In particular, each scan was performed over 80 s with a rotation time of 19 s per revolution. The flat detector provides a framerate of 100 fps (frames per second) allowing for phase-correlated imaging. All image reconstructions were performed using a low-dose phase-correlated reconstruction method; that is, all reconstructions show a distinct cardiac and respiratory state of the animal to prevent degradation of image quality due to motion artifacts [20] and, hence, degradation of derived functional and morphological parameters [21]. In particular, all reconstructions performed herein present an exhale respiratory state and a diastolic cardiac state, while the reconstructions incorporate 10% of the data in the corresponding motion cycle. The required ECG data was obtained via small electrodes attached to the animal's paw and the respiratory signal was derived using a pneumatic pillow (Small Animal Instruments, Stony Brook, USA). To allow for repetitive image acquisitions, the scan protocol was optimized to minimize radiation dose. Each measurement within the 250 min period comprises a radiation dose of 50 mGy accumulating to a total dose of 600 mGy. The X-ray tube was operated using a tube voltage of 80 kV and a tube current of 50 mA. Signal enhancement was measured in regions of interest (ROIs) placed in different tissues, namely, the left ventricle (corresponding to vasculature), myocardium, liver (avoiding larger intrahepatic vessels), spleen, kidney (renal cortex), brain, muscle (represented by the gastrocnemius muscle), and brown adipose tissue (represented by the suprascapular adipose tissue).

Signal enhancement in the myocardium of mice with myocardial infarction (*n* = 5) at 210 min postinjection (p.i.) of the contrast agent was utilized for subsequent quantification of infarct size.

For determination of the minimum dose of ExiTron MyoC 8000 that enables effective imaging of the myocardium, healthy mice (*n* = 5) were each administered with a different dose of the contrast agent (210, 420, 630, 840, and 1050 mg I/kg body weight, corresponding to 25, 50, 75, 100, and 125 *μ*L per 25 g mouse, resp.). The dose-dependent signal enhancement was measured at 15 min postinjection in the blood (left ventricle) as well as at 210 min postinjection in the myocardium.

### 2.3. Histology

After imaging, the mice were immediately sacrificed and their hearts were excised, flushed with phosphate buffered saline (PBS), and embedded in paraffin. Transverse slices of 10 *μ*m thickness were prepared and stained using Masson's Trichrome [22], which stains viable muscle red and collagen green allowing for improved discrimination between healthy myocardium and the collagenous scar of the infarcted region [23]. To quantify infarct size in histological stains and reconstruct three-dimensional images we follow a protocol proposed recently [24]. In particular, we define the infarct size as the relative amount of infarcted mid-myocardial contour area (MCA) with MCA being the area of the myocardial wall enclosing the left ventricle measured between endocardium and epicardium. This method was chosen as it inherently compensates for a homogeneous shrinking due to the paraffin embedding and staining procedure, a thinning of the infarcted area, and an increase in healthy myocardium to compensate for the infarction.

## 3. Results and Discussion

**Figure 1:**
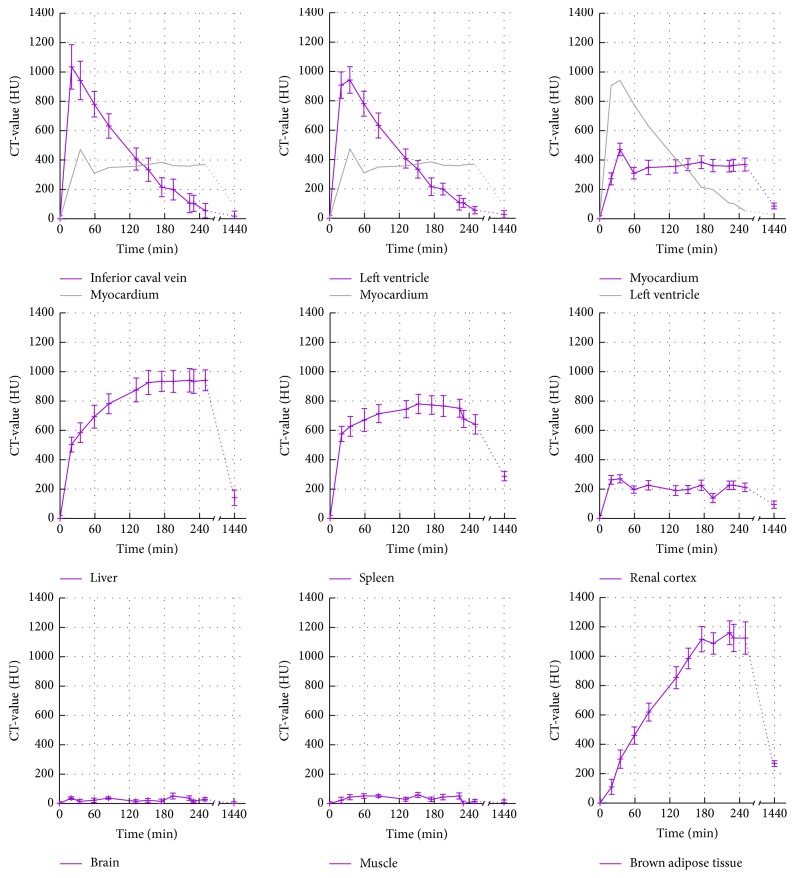
Time-dependent signal enhancement, that is, baseline-corrected CT-values, in different tissues of healthy C57BL/6J mice (*n* = 10) before and after injection of ExiTron MyoC 8000 at a dose of 1050 mg I/kg body weight.

In order to investigate the kinetics of ExiTron MyoC 8000, signal enhancements, that is, baseline-corrected CT-values, in tissues of healthy mice were measured over a period of 250 min after injection of the contrast agent at a dose of 1050 mg I/kg body weight with an additional scan after 24 h (Figure 1). The time-enhancement curve of the left ventricle illustrates that the contrast agent has a long blood half-life of ~2 h, with a peak enhancement of ~950 HU observed immediately after injection. At 250 min postinjection the signal enhancement in the blood has nearly reached a baseline value denoting that at this time point the contrast agent has been practically cleared from the blood. If one considers an enhancement of 200 HU sufficient for functional imaging, for example, for evaluation of the left ventricular ejection fraction through measurement of the blood volume in the left ventricle during systole and diastole, such experiments can be conducted over a time period of ~3 h after injection of the contrast agent at the mentioned dose. This extended imaging time window allows for the implementation of challenging imaging protocols, even in cases where a less rapid micro-CT system is in operation.

**Figure 2:**
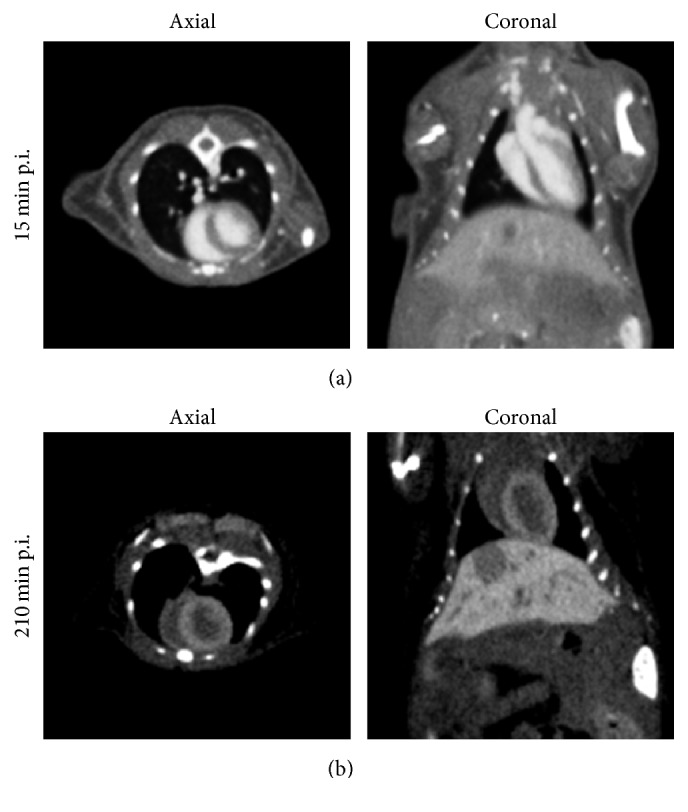
Axial and coronal micro-CT reconstruction images obtained at 15 min ((a), *C*/*W* = 500/1500 HU) and 210 min ((b), *C*/*W* = 500/850 HU) postinjection of ExiTron MyoC 8000 in healthy C57BL/6J mice at a dose of 1050 mg I/kg body weight. In the early phase the blood is highly contrasted allowing for functional cardiac imaging, whereas in the later phase vessel contrast is diminished enabling delineation of the myocardium.

Similar to the signal enhancement in the blood, the signal enhancement in the myocardium rapidly increases following injection of ExiTron MyoC 8000, an effect that has also been reported for a different nanoparticulate contrast agent [5, 6]. The mechanism of uptake of ExiTron MyoC 8000 by the myocardium is as yet unknown but is believed to be related to nanoparticle surface properties rather than to nanoparticle size. The peak enhancement in the myocardium is observed at 20 min postinjection of the contrast agent but is likely overestimated due to an overexposure artifact resulting from the strong contrast in the adjacent ventricles. Thereafter, the signal enhancement in the myocardium is found to remain constant throughout, measuring ~350 HU. As a result, ExiTron MyoC 8000 enables not only functional cardiac imaging during the first 2 h of the blood pool phase but also imaging of the myocardium at a later phase. Micro-CT images obtained during these two phases highlight the differences in signal enhancement within the various tissues (Figure 2). Further reconstruction results and animations can be found in the Supplementary Material (see Supplementary Material available online at https://doi.org/10.1155/2017/2617047). The gray value scaling of all reconstructions presented herein is described as gray level center (*C*) and width (*W*) measured in Hounsfield Units (HU), as commonly used in CT. The images therefore only show the fraction of CT-values that lie within *C* ± 0.5 · *W* with all CT numbers below the lower limit of the window width displayed as black and all those above the upper limit displayed as white. The vessels, ventricles, and aortic arch can be easily discriminated from surrounding tissues at 15 min postinjection, whereas vessel contrast is diminished at 210 min postinjection enabling delineation of the myocardium.

Besides providing signal enhancement in the blood and myocardium, ExiTron MyoC 8000 also results in the enhancement of other tissues (Figure 1). A strong signal enhancement is observed in both liver (about 900 HU) and spleen (about 700 HU) indicating that these are the main excretory organs. Since signal enhancement is also observed in the kidneys, albeit at a much lower extent (about 250 HU), the contrast agent is also eliminated by renal clearance. In brown adipose tissue (BAT), signal enhancement progressively increases with time, an effect that has been previously observed with a different nanoparticulate CT agent [5, 6] showing a maximum enhancement of about 1100 HU measured at 210 min postinjection. As expected, the muscle and brain tissues do not show any significant uptake of the contrast agent. At the 24-hour time point (1440 min) the signal enhancement curves of the myocardium, liver, spleen, kidney, and BAT converge with the baseline indicating that the contrast agent is almost eliminated from the body, offering the possibility of multiple injections.

**Figure 3:**
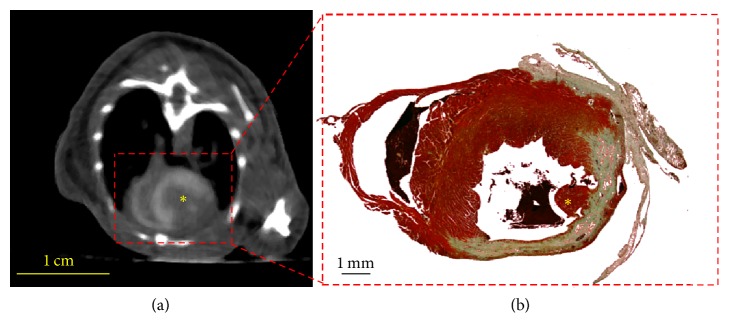
Axial micro-CT reconstruction image of mouse with myocardial infarction 210 min after injection of ExiTron MyoC 8000 at a dose of 1050 mg I/kg body weight ((a), *C*/*W* = 350/700 HU) and corresponding microscopy image of histological section depicting viable tissue in red and infarct scar as well as local collagenous tissue in green (b). Note the viable papillary muscle (yellow asterisk), which can be identified in both the micro-CT reconstruction image and the microscopy image.

**Figure 4:**
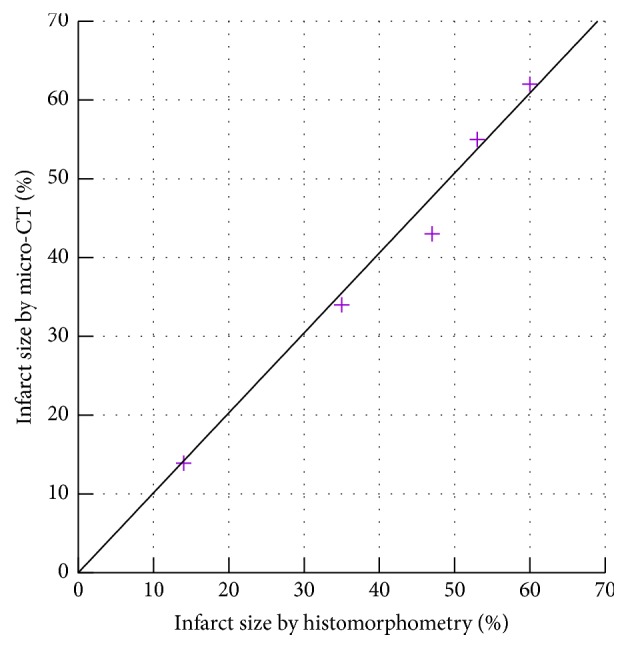
Comparison of infarct size obtained via micro-CT reconstruction images and via histomorphometry shows a significantly strong correlation (*R*^2^ = 0.98).

Since imaging at 210 min postinjection allows for effective visualization of the myocardium, this time point was selected for subsequent studies in a mouse model of myocardial infarction. Through micro-CT reconstruction images, infarct sizes were quantified and compared to those obtained via histopathology. A qualitative comparison of the micro-CT reconstruction images and microscopy images of histological sections depicts that the contrast agent is homogeneously distributed in the healthy myocardium but is not taken up by areas of myocardial infarction (Figure 3). The infarct sizes obtained via imaging as well as via histology vary in the range of 14 % and 60 % (Figure 4) and are similar to those previously reported [24]. A quantitative comparison of infarct sizes obtained via the two methods shows a significantly strong correlation (*R*^2^ = 0.98). This demonstrates that micro-CT using ExiTron MyoC 8000 allows for noninvasive studies of cardiac processes without the need of sacrificing the animals under investigation for histomorphometry.

**Figure 5:**
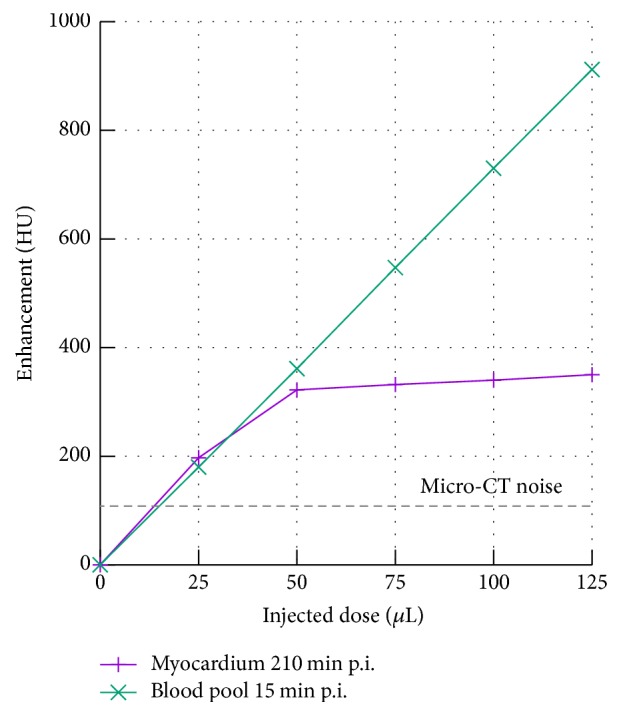
Signal enhancement in the blood at 15 min postinjection and in the myocardium at 210 min postinjection at different doses of the injected contrast agent. The dashed line indicates the noise measured in soft tissue given the imaging protocol described herein and might be different for other scan protocols or system configurations.

So as to determine the minimum contrast agent dose that enables effective imaging of the myocardium, healthy C57BL/6J mice (*n* = 5) were each injected with a different dose of the contrast agent (Figure 5). The animals were scanned initially at 15 min postinjection to monitor the dose-dependent contrast in the blood (left ventricle) and then again at 210 min postinjection to measure the dose-dependent signal enhancement in the myocardium. As expected, the signal enhancement in the blood is linearly proportional to the amount of contrast agent injected. However, the signal enhancement in the myocardium first increases with increasing dose (from 200 HU at 210 mg I/kg body weight to 350 HU at 420 mg I/kg body weight) but then remains constant preventing increased signal-to-noise ratios to be achieved at higher doses. Nevertheless myocardial imaging can be conducted using ExiTron MyoC 8000 at a dose as low as 420 mg I/kg body weight (corresponding to a volume of 50 *μ*L/25 g mouse) in the used imaging setup, which is particularly beneficial for weak animals with severe cardiac pathologies. As observed in Figure 5, imaging at this low dose of contrast agent results in a blood pool signal enhancement of 350 HU at 15 min postinjection, which we believe is still sufficient for functional and morphological studies, for example, estimation of the left ventricular ejection fraction, end-systolic volume or end-diastolic volume. This is supported by the fact that, with the used scan protocol, the noise in soft tissue measures ~100 HU (dashed line in Figure 5), demonstrating that the blood pool enhancement can be easily discriminated from background noise, even at this low contrast agent dose. Note that the minimum required contrast agent dose is a function of the scanner setup and acquisition protocol, particularly the used tube voltage and overall radiation dose.

## 4. Conclusion

In this study, a micro-CT method using the innovative contrast agent ExiTron MyoC 8000 was developed for longitudinal* in vivo* monitoring of cardiac processes in small animals. The low-dose phase-correlated micro-CT reconstruction method provides images with superior spatial and temporal resolution. Due to its high iodine concentration (210 mg I/mL), long blood half-life (~2 h), and accumulation in healthy myocardium, ExiTron MyoC 8000 enables not only functional cardiac imaging but also imaging of the myocardium, even at a very low dose (50 *μ*L per 25 g mouse). Using a mouse model of myocardial infarction, we proved that the approach offers an accurate and reproducible method for the* in vivo* quantification of infarct size. The low injection volume of the contrast agent coupled with the low radiation dose of the micro-CT method renders this technique particularly beneficial for longitudinal studies of weak animals with severe cardiac pathologies.

## Supplementary Material

Additional reconstructions showing healthy mice and such with myocardial infarction.

## References

[1] Ritman E. L. (2011). Current Status of Developments and Applications of Micro-CT. *Annual Review of Biomedical Engineering*.

[2] Clark D. P., Badea C. T. (2014). Micro-CT of rodents: State-of-the-art and future perspectives. *Physica Medica*.

[3] Socher M., Kuntz J., Sawall S., Bartling S., Kachelrieß M. (2014). The retrobulbar sinus is superior to the lateral tail vein for the injection of contrast media in small animal cardiac imaging. *Laboratory Animals*.

[4] Kuntz J., Dinkel J., Zwick S. (2010). Fully automated intrinsic respiratory and cardiac gating for small animal CT. *Physics in Medicine and Biology*.

[5] Detombe S. A., Dunmore-Buyze J., Drangova M. (2012). Evaluation of eXIA 160 cardiac-related enhancement in C57BL/6 and BALB/c mice using micro-CT. *Contrast Media & Molecular Imaging*.

[6] Ashton J. R., Befera N., Clark D. (2014). Anatomical and Functional Imaging of Myocardial Infarction in Mice Using Micro-CT and eXIA 160 Contrast Agent: Cardiac MicroCT for Imaging Myocardial Infarction. *Contrast Media Molecular Imaging*.

[7] Mannheim J. G., Schlichthaerle T., Kuebler L. (2016). Comparison of small animal CT contrast agents. *Contrast Media & Molecular Imaging*.

[8] Martiniova L., Schimel D., Lai E. W., Limpuangthip A., Kvetnansky R., Pacak K. (2010). In vivo micro-CT imaging of liver lesions in small animal models. *Methods*.

[9] Choukèr A., Lizak M., Schimel D. (2008). Comparison of Fenestra VC contrast-enhanced computed tomography imaging with gadopentetate dimeglumine and ferucarbotran magnetic resonance imaging for the in vivo evaluation of murine liver damage after ischemia and reperfusion. *Investigative Radiology*.

[10] Boll H., Figueiredo G., Fiebig T. (2013). Comparison of Fenestra LC, ExiTron nano 6000, and ExiTron nano 12000 for Micro-CT imaging of liver and spleen in mice. *Academic Radiology*.

[11] Boll H., Nittka S., Doyon F. (2011). Micro-CT based experimental liver imaging using a nanoparticulate contrast agent: A longitudinal study in mice. *PLoS ONE*.

[12] Gargiulo S., Greco A., Gramanzini M. (2012). PET/CT imaging in mouse models of myocardial ischemia. *Journal of Biomedicine and Biotechnology*.

[13] Roelants V., Delgaudine M., Walrand S. (2012). Myocardial infarct size quantification in mice by SPECT using a novel algorithm independent of a normal perfusion database. *EJNMMI Research*.

[14] Chapon C., Herlihy A. H., Bhakoo K. K. (2008). Assessment of myocardial infarction in mice by Late Gadolinium Enhancement MR imaging using an inversion recovery pulse sequence at 9.4T. *Journal of Cardiovascular Magnetic Resonance*.

[15] Coolen B. F., Paulis L. E. M., Geelen T., Nicolay K., Strijkers G. J. (2012). Contrast-enhanced MRI of murine myocardial infarction - Part II. *NMR in Biomedicine*.

[16] Mähler M., Berar M., Feinstein R. (2014). FELASA recommendations for the health monitoring of mouse, rat, hamster, guinea pig and rabbit colonies in breeding and experimental units. *Laboratory Animals*.

[17] Wang J., Bo H., Meng X., Wu Y., Bao Y., Li Y. (2006). A simple and fast experimental model of myocardial infarction in the mouse. *Tex Heart Inst J*.

[18] Kolk M. V. V., Meyberg D., Deuse T. (2009). LAD-ligation: a murine model of myocardial infarction.. *Journal of visualized experiments : JoVE*.

[19] Fessi H., Piusieux F., Devissaguet J. P., Ammoury N., Benita S. (1989). Nanocapsule formation by interfacial polymer deposition following solvent displacement. *International Journal of Pharmaceutics*.

[20] Sawall S., Bergner F., Lapp R. (2011). Low-dose cardio-respiratory phase-correlated cone-beam micro-CT of small animals. *Medical Physics*.

[21] Maier J., Sawall S., Kachelrieß M. (2014). Assessment of dedicated low-dose cardiac micro-CT reconstruction algorithms using the left ventricular volume of small rodents as a performance measure. *Medical Physics*.

[22] Zhao X., Wu J., Gray C. D. (2015). Optical projection tomography permits efficient assessment of infarct volume in the murine heart postmyocardial infarction. *American Journal of Physiology-Heart and Circulatory Physiology*.

[23] Bishop J. E., Greenbaum R., Gibson D. G., Yacoub M., Laurent G. J. (1990). Enhanced deposition of predominantly type I collagen in myocardial disease. *Journal of Molecular and Cellular Cardiology*.

[24] Stegger L., Hoffmeier N. A., Schafers K. P. (2006). Accurate noninvasive measurement of infarct size in mice with high-resolution PET. *Journal of Nuclear Medicine*.

