# Supplementary material for: In Vivo Quantification of Myocardial Infarction in Mice Using Micro-CT and a Novel Blood Pool Agent
Source: Contrast Media Mol Imaging. 2017 Oct 16;2017:2617047. doi: 10.1155/2017/2617047 (PMC5662822; doi:10.1155/2017/2617047)
Supplement: Supplementary file 1 — Additional reconstructions showing healthy mice and such with myocardial infarction. [file 2617047.f1.pptx]

## Slide 1
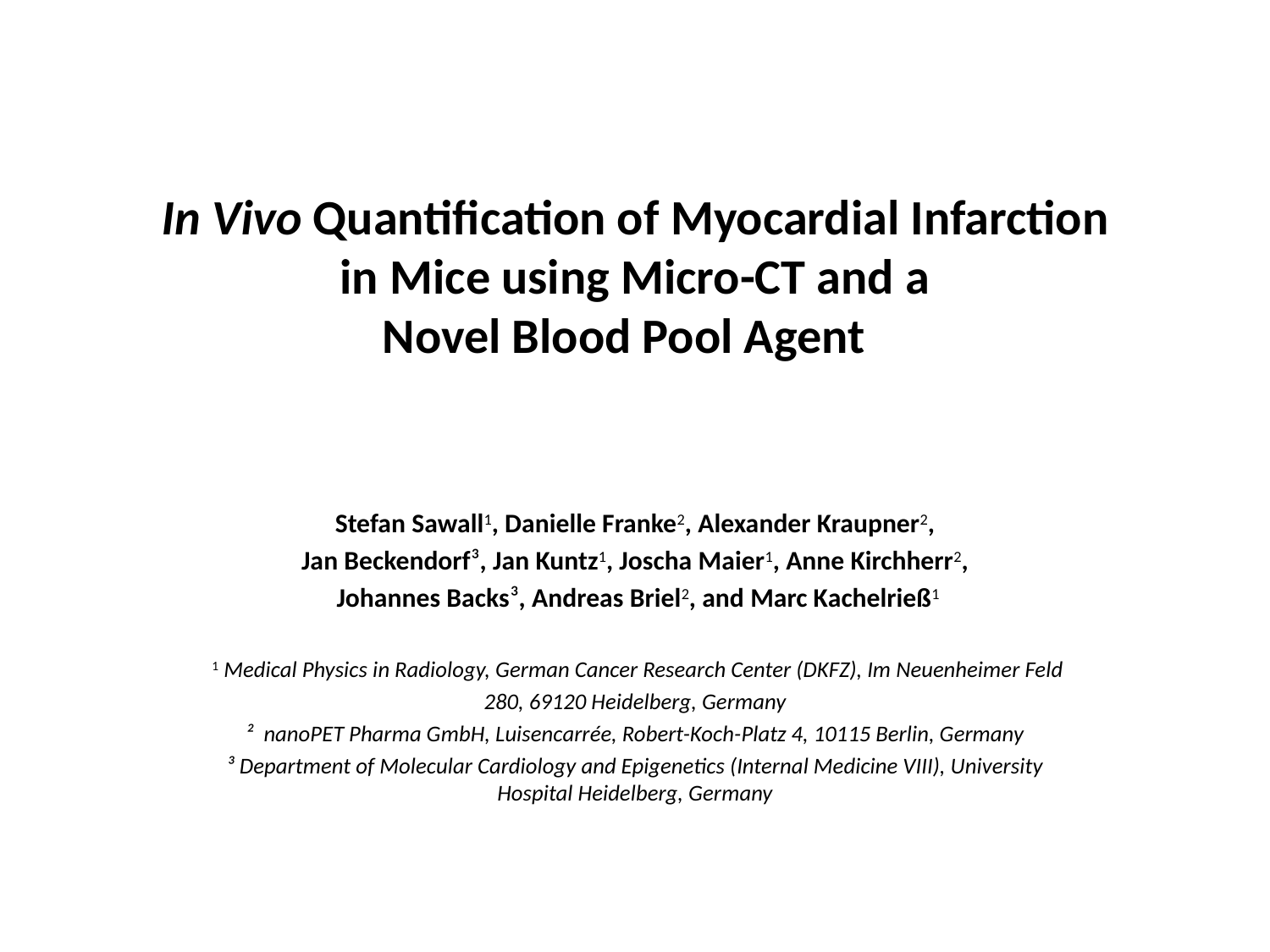

# In Vivo Quantification of Myocardial Infarction in Mice using Micro-CT and a Novel Blood Pool Agent
Stefan Sawall1, Danielle Franke2, Alexander Kraupner2,
Jan Beckendorf³, Jan Kuntz1, Joscha Maier1, Anne Kirchherr2,
 Johannes Backs³, Andreas Briel2, and Marc Kachelrieß1
 1 Medical Physics in Radiology, German Cancer Research Center (DKFZ), Im Neuenheimer Feld 280, 69120 Heidelberg, Germany
² nanoPET Pharma GmbH, Luisencarrée, Robert-Koch-Platz 4, 10115 Berlin, Germany
³ Department of Molecular Cardiology and Epigenetics (Internal Medicine VIII), University Hospital Heidelberg, Germany

## Slide 2
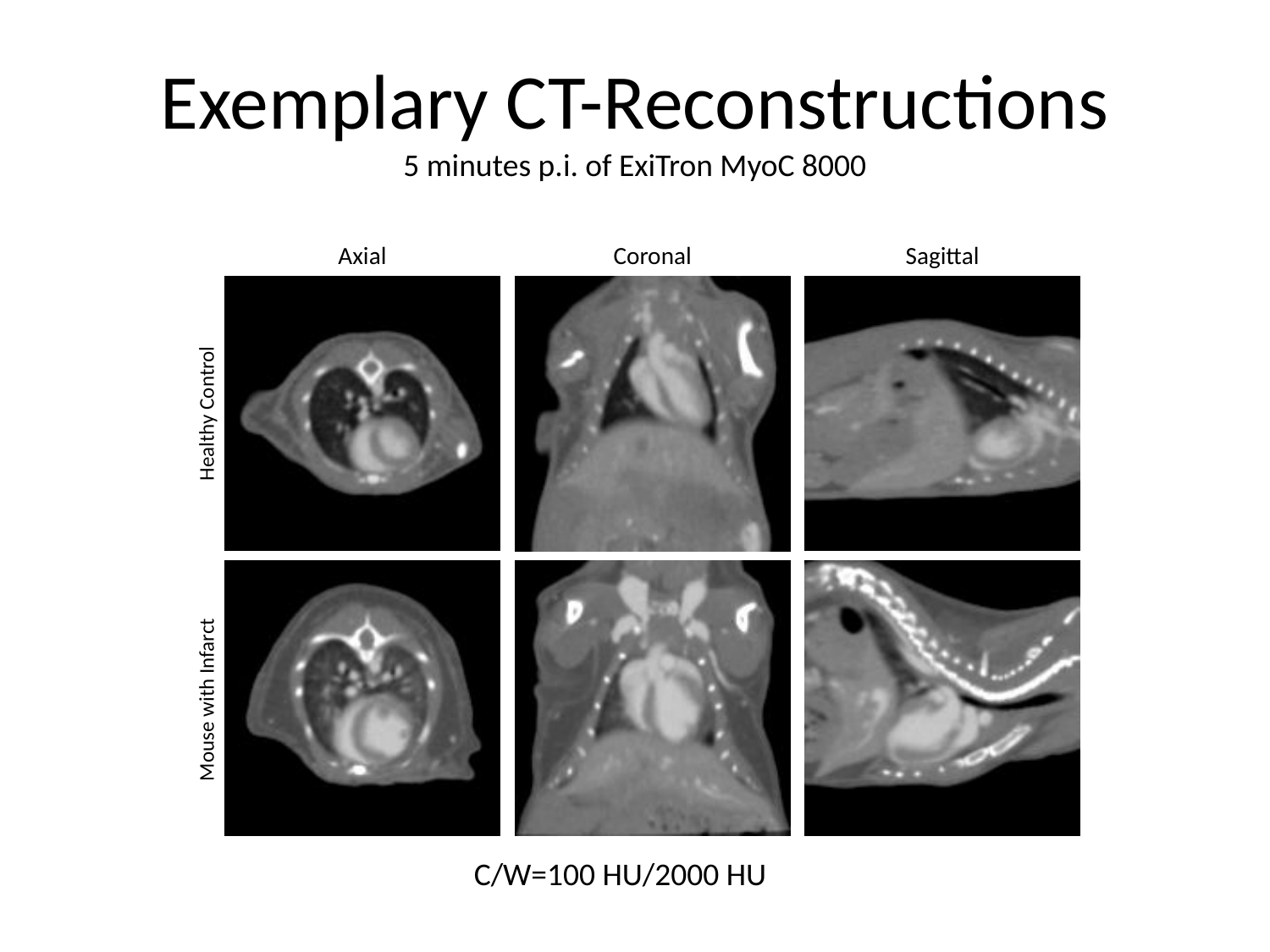

# Exemplary CT-Reconstructions 5 minutes p.i. of ExiTron MyoC 8000
Axial
Coronal
Sagittal
Healthy Control
Mouse with Infarct
C/W=100 HU/2000 HU

## Slide 3
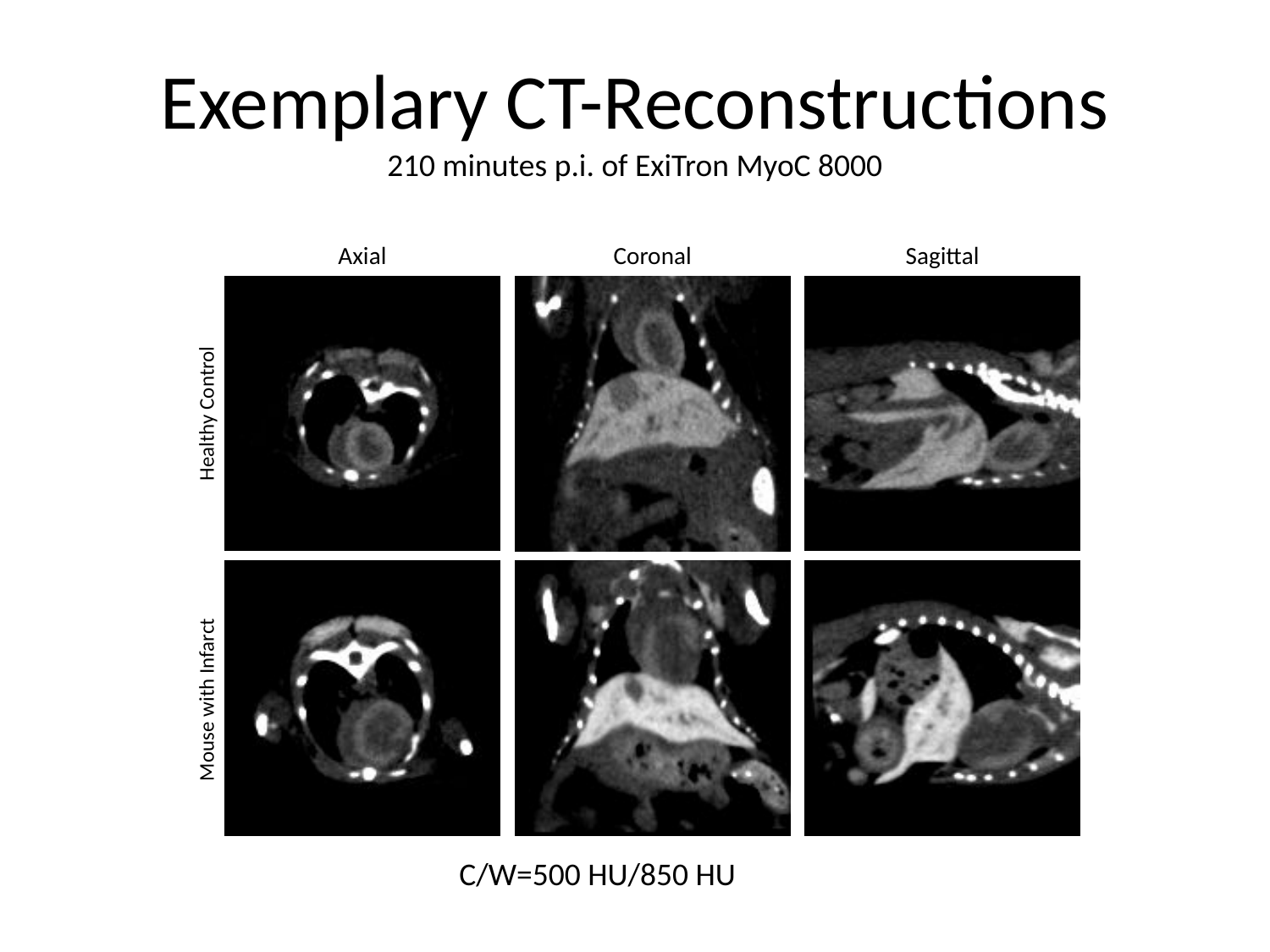

# Exemplary CT-Reconstructions 210 minutes p.i. of ExiTron MyoC 8000
Axial
Coronal
Sagittal
Healthy Control
Mouse with Infarct
C/W=500 HU/850 HU

## Slide 4
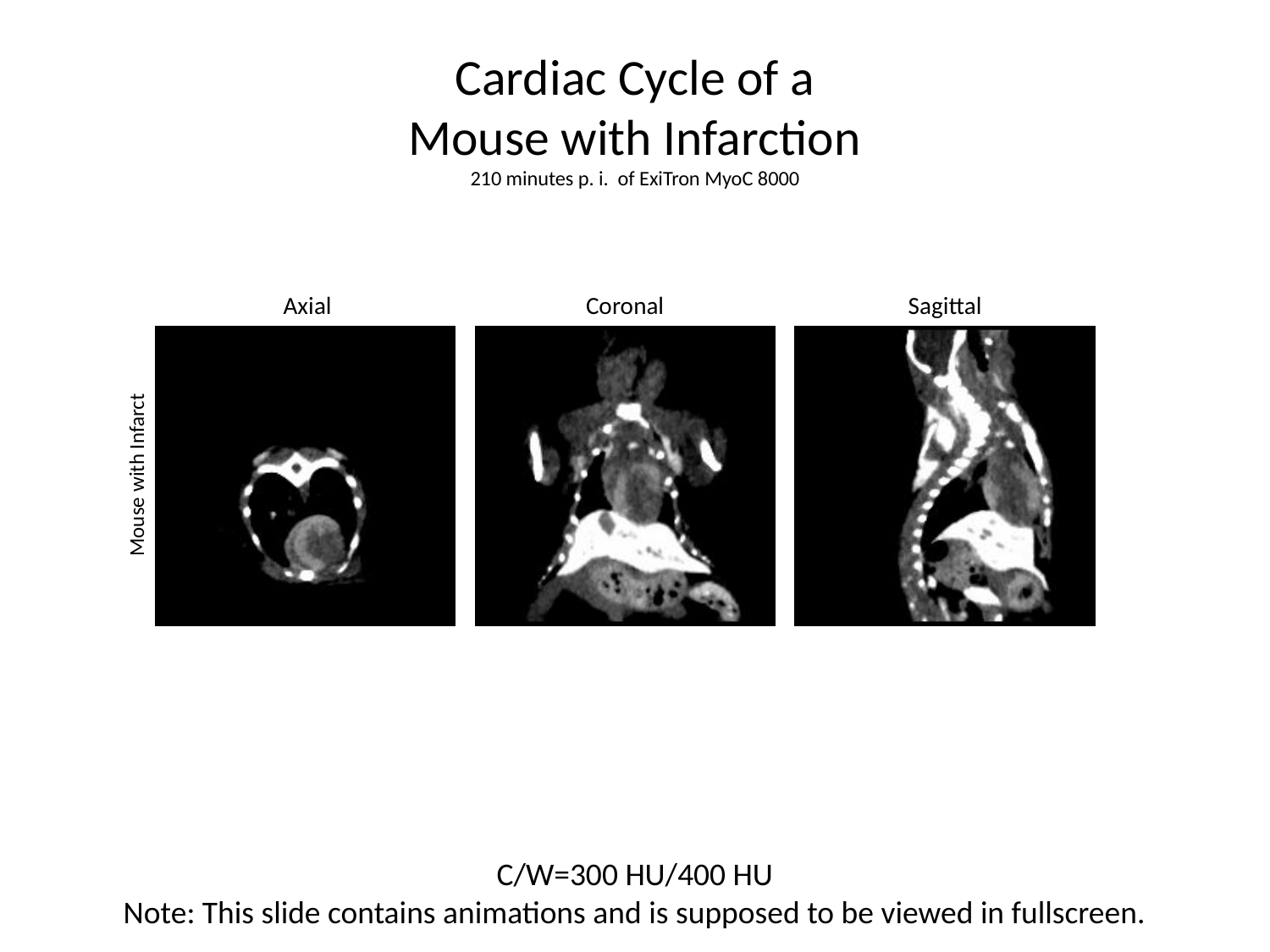

# Cardiac Cycle of a Mouse with Infarction 210 minutes p. i. of ExiTron MyoC 8000
Axial
Coronal
Sagittal
Mouse with Infarct
C/W=300 HU/400 HU
Note: This slide contains animations and is supposed to be viewed in fullscreen.
